# A prevalence and molecular characterization of novel pathogenic strains of *Macrococcus caseolyticus* isolated from external wounds of donkeys in Khartoum State –Sudan

**DOI:** 10.1186/s12917-022-03297-2

**Published:** 2022-05-25

**Authors:** Dania E. Ali, Mushal Allam, Hisham N. Altayb, D. Mursi, M. A. Adalla, N. O. Mohammed, Mona A. M. Khaier, Manal H. Salih, Sarah Abusalab, M. A. Abbas

**Affiliations:** 1 Animal Resources Research Corporation, Sudan Academy of Science, Khartoum, Sudan; 2 Department of Genetics and Genomics, United Arab Emirates University, Al Ain, United Arab Emirates; 3 Biochemistry Department, Faculty of Sciences, King Abdulaziz University, Jeddah, 21452 Saudi Arabia; 4 Central Laboratory, Ministry of Higher Education and Scientific Research, Khartoum, Sudan; 5 Central Veterinary Research Laboratory, Department of Biological Products, Animal Resources Research Corporation, Khartoum, Sudan; 6 Ahfad Center for Science and Technology, Ahfad University for Women, Omdurman, Sudan; 7 Department of Molecular Biology and Bioinformatics, University of Bahri, Khartoum, Sudan; 8 Central Veterinary Research Laboratory, Department of Pathology, Animal Resources Research Corporation, Khartoum, Sudan; 9 Department of Preventive Medicine and Veterinary Public Health, Faculty of Veterinary Medicine, University of Khartoum, Khartoum, Sudan; 10 Department of Microbiology, Faculty of Veterinary Medicine, University of Khartoum, Khartoum, Sudan

**Keywords:** *Macrococcus caseolyticus*, External wounds, Whole-genome sequencing, Bioinformatics analysis, Antimicrobial sensitivity test, Mice model

## Abstract

A pathogenic strain of *Macrococcus caseolyticus* (*M. caseolyticus*) was isolated from wounds infection during an investigation on donkeys in Khartoum State. (122) samples were collected from external wounds (head, abdomen, back and leg) during different seasons. One isolate (124B) was identified using whole-genome sequence analysis. RAST software identified 31 virulent genes of disease and defense, including methicillin-resistant genes, TatR family and ANT(4′)-Ib. Plasmid rep22 was identified by PlasmidFindet-2.0 Server and a CRISPR. MILST-2.0 predicted many novel alleles. NCBI notated the genome as a novel *M. caseolyticus* strain (*DaniaSudan)*. The MLST_−_tree_−_V1 revealed that *DaniaSudan* and KM0211a strains were interrelated. Strain *DaniaSudan* was resistant to ciprofloxacin, ceftazidime, erythromycin, oxacillin, clindamycin and kanamycin. Mice modeling showed bacteremia and many clinical signs (swelling, allergy, wounds, and hair loss). Enlargement, hyperemia, adhesions and abscesses were observed in many organs.

**Constructive conclusion**

The prevalence of the strain was 4.73%, with significant differences between collection seasons and locations of wounds. A highly significant association between doses (10^5^ CFU/ml, 10^2^ CFU/ml, Intra-peritoneum and sub-cutaneous) and swelling, developing of allergy and loss of hair (*p* = 0.001, *p* = 0.000 and *p* = 0.005) respectively were seen.

This result represents the first report of pathogenic strains of *M. caseolyticus* worldwide.

**Supplementary Information:**

The online version contains supplementary material available at 10.1186/s12917-022-03297-2.

## Introduction

Donkey or Ass (*Equus ansinus*) descended from the African and Asian wild Asses and was assumed the first domesticated member of the Equidae family [1]. In rural and urban areas of Sudan, donkeys play a critical role in supporting low-income families. Thus, their protection in good health is essential. The world’s donkey population is about 44 million [2]. In 2017, the population of donkeys in Sudan was 7,597,458; 0.10% of them were found in Khartoum State (Statistical Bulletin for Animal Resources, 2017). In Khartoum State, 30.8% of investigated animals suffered from different wounds, while 61.9% of donkeys in Ethiopia had wounds [3, 4]. Overloads and excessive work were important factors that led to stress and injury [5]. It was reported that disease and health problems affect working equids and their productivity [6]. Hence, it is important to study the causative agents of the donkey’s wound infections.

Dania 2017, reported that in Khartoum State, 30.8% of investigated animals were suffering from different wounds, 59.4% of the infected animals suffered from primary wound infections, including subcutaneous abscesses, folliculitis, lymphangitis, equine staphylococcal dermatitis and thrush, 20.5% of wounds of donkeys were due to fistulous withers, glanders, pasteurella infection, listeriosis, sleepy foal disease and strangles [6].

*M. caseolyticus* was initially named *Micrococcus caseolyticus* by Evans in 1916. It was then renamed *Staphylococcus caseolyticus* by Schleifer in 1982 [7]. It received its current designation in 1998 by Kloos [8]. *M .caseolyticus* is a gram-positive bacterium, catalase-positive, oxidase-positive and grows aerobically. It is alkaline phosphatase, urease and a weak reaction to esculin hydrolysis. Acid produces from maltose and weekly from sucrose [9].

Some strains have acquired antibiotic resistance mechanisms identical or similar to those found in staphylococci, such as *cfr*-mediated multidrug resistance and *mecB*-mediated methicillin resistance [10]. *M. caseolyticus* strains JCSC5402, JCSC7096, and JCSC7528 carry the *mecAm* gene while being negative in methicillin-resistant [11]. The *mecB* was found in *M. caseolyticus* either within a *SCCmec*-like element or carried on a plasmid [10–12]. *M. caseolyticus* subsp. *Hominis* (type strain CCM 7927 T = DSM 103682 T) was isolated from various human clinical materials [9].

This study aimed to isolate, and identify *M. caseolyticus* from donkey’s wounds in Khartoum State, determine the prevalence and sensitivity of the isolated bacterium to different antimicrobial drugs, and study the toxicity and pathogenicity of the organism in mice model.

## Material and methods

### Experimental design

Three hundred twelve (312) donkeys were investigated for the presence of external wounds from Dec. 2015 to Aug. 2016. Isolates were collected in winter, summer and autumn seasons and coded > 100, < 100 and < 200, respectively. The fieldwork was carried out in Khartoum State according to the gathering-sits of the last population. A structured direct format was developed and data was collected from animal owners or users after explaining the study’s objective. The age of animals was estimated based on the observation of the animal’s front teeth (Incisors) [13] and categorized into> 10 years and < 10 years. Samples were taken from body lesions in back, abdominal, head and leg sores. The duration of work was recorded to > 8 hours and < 8 hours.

### Isolation and identification of the bacterial agent

One hundred twenty-two (122) swab samples were collected from wounds secretions after the owner’s verbal consent. The swabs (Copan) were preserved in ice and transported to the laboratory within 4-6 hours. Samples were streaked onto fresh Blood Agar plate medium (Oxoid) and incubated aerobically at 37 °C for 1-3 days. Isolated bacteria were purified by repeated sub-cultures in Blood Agar Plates. The identification of isolates was carried out according to Barrow and Feltham [14]. The study was under the standard biosecurity and institutional safety procedures of Animal resources research cooperation Khartoum, Sudan.

### Polymerase Chain Reaction (PCR)

Only 23 isolates that were Gram-positive and negative to the oxidation fermentation test were tested by PCR. They were cultured on fresh Blood agar (Oxoid) and incubated aerobically at 37 °C for 24 hours. Genomic DNA was extracted by boiling. Three to five colonies were transferred into a 1.5 ml sterile Eppendorf tube containing 50 μl distilled water. The mixture was homogenized and transferred to a boiling water bath for 15 min. Then the mixture was cooled in ice for 2 min and centrifuged at 13.000 rpm for 5 min [15]. Five microlitre of the supernatant were collected and used directly for PCR. A set of universal 16S rRNA primer F (5^\^CCAGCAGCCGCGGTAATACG3^\^) and R (5^\^ATCGGYACCTTGTTACGACTTC3^\^) were selected from a published sequence [16]. Five microlitre of genomic DNA from each isolate was added to the PCR mixture of 12.5 μl green master mix and 3.2 pmol of each primer, dH_2_O was added to reach a total volume of 25 μl. The PCR reaction was run on (Peqlab) thermo-cycler. The following parameters of the program were used with modification: initial denaturation step at 94 °C for 5 min; 35 cycles of denaturation at 94 °C for 1 min, annealing at 55 °C for 1 min and extension at 72 °C for 2 min and a final extension at 72 °C for 10 min [16]. The PCR products were was stained with Ethidium Bromide (1 μg/ml) and visualized with a short-wave ultraviolet light stored at 8^o^ C until required for electrophoreses. Five microlitre of each PCR product were loaded in the 1.5% agarose gel wells. The gel electrophoresis was run in Ix Tris-acetate buffer (TAE) at 100 V for 30 minutes. The gel [17]. Bands were compared with the standard DNA ladder. For sequencing the PCR products were sent to Macrogen Inc. Seoul, South Korea. The alignment was done with BLAST at The National Center for Biotechnology Information (www.ncbi.nlm.nih.gov).

### Whole-genome sequencing and bioinformatics analysis

One ampoule of lyophilized isolate (124B) was opened and reconstituted in 4.5 ml of fresh brain heart infusion broth (BHIB) (Oxoid) and incubated aerobically at 37^0^ C for 24 hours. The tube was centrifuged at 100 rpm. Genomic DNA was extracted using a commercial DNA purification kit (innuPREP DNA Mini Kit, Analytik Jena, Germany) to obtain high quality and quantity. Forty microlitre of genomic DNA of isolate 124B were sent to BGI Company in China for Whole-genome sequencing by illumine Hiseq4000. Assembly was done by unicycle assemplyv.0.4.4 with genome coverage 100.0xs.Mauve [18], which were multiple alignments of whole-genomes and alignment functions, was used to order the contigs (http://asap.ahabs.wisc.edu/mauve/). Annotation was done by Annotation Pipeline NCBI Prokaryotic Genome Annotation Pipeline; Annotation +method (Best-placed reference protein set and GeneMarks+ Annotation software revision;4.6) and an automated web-based tool, RAST [19]. The annotated genes were exported from the RAST server into an excel table and manually compared for genomic features (http://rast.nmpdr.org/). The graphical circular map of those genomes was made by CGView server. Center for Genomic Epidemiology and BLAST were used to determine the specific genes as gene resistance determinants, plasmid and MLST [20]. Phylogenetic analysis of strain daniaSudan was done by NCBI Tree Viewer (Tree Viewer 1.17.5), which is software using the neighbor-joining method [21] and calculated by Kimura’s two-parameter model [22].

### The prevalence of strain DaniaSudan

Following identification of isolate 124B by WGS, the nine PCR product sequences were aligned with isolate 124B as control and strain JCSC5402 as a reference strain by BLAST. The prevalence of the strain was calculated from the total isolates.

### Sensitivity test to antimicrobial susceptibility testing

Isolate 124B was cultured in Brain-Heart infusion broth (Oxoid) aerobically at 37^0^ C for 24 hours. A sterile swab was dipped in the suspension of the bacterial growth and cultured onto the entire surface of Muller-Hinton agar (Oxoid). The following antibiotic discs (Bioanlye) were applied on the surface of bacterial Muller-Hinton agar: Ampicillin (10 μg), cefoxitin (30 μg), ceftazidime (10 μg), cephalothin (30 μg), ciprofloxacin (5 μg), clindamycin (2 μg), gentamicin (10 μg), chloramphenicol (30 μg), imipenem (10 μg), neomycin (10 μg), novobiocin (5 μg), oxacillin (1 μg), penicillin G (1 IU), cotrimoxazole (25 μg), tetracycline (30 μg) and vancomycin (30 μg). Then the organism under test was aerobically incubated at 37^0^ C for 24 hours. The inhibitory zones diameters were measured and translated to resistance levels (susceptible-intermediate-resistance) in accordance with the Performance Standards for Antimicrobial Disc Susceptibility tests [23–25]; EUCAST, http://www.eucast.org.

### Pathogenesis study of strain DaniaSudan

#### Experimental animals

Forty-six (46) mice of average weight 25 g were purchased from the department of small laboratory animals at Central Veterinary Laboratory, Khartoum, Sudan. Mice were housed in a temperature and light-controlled environment with free food and sterile water access. After adaptation to the light-dark cycle for 1 week, the experiment was started. Isolate 124B was cultured in BHI agar (Oxoid) aerobically at 37^0^ C for 24 hours. Two dilutions (10^5^ CFU/ml and 10^2^ CFU/ml) were prepared for sup-cutaneous and intra-peritoneum injection. One millilitre of the supernatant was prepared for intra-peritoneum injection [26]. Four mice were injured by a sterile needle in the head, back, abdomen and leg and cultured with the organism under test with disposable swabs.

The study was carried out in compliance with the ARRIVE guidelines.

#### Mice- strain DaniaSudan-infection model

Mice were housed within the premises of the lab at Soba, Khartoum in adlib fed**.** The mice were divided into 7 groups (Table 1). The temperatures of mice under test were measured before and every 12 hours after injection for 7 days. Then the mean temperatures of all groups were calculated. Mice under test were observed, and postmortem was conducted after 7 days. Smears from collected organs were cultured and identified as attested organisms.

**Table 1:** *M. caseolyticus* strain DaniaSudan injection at different site in mice

Group Number	Number of mice	Infective dose	Route of infection
Group 1	7	10^5^ CFU/ml	Sub-cutaneous
Group 2	7	10^5^ CFU/ml	Intra-peritoneum
Group 3	7	10^2^ CFU/ml	Sub-cutaneous
Group 4	7	10^2^ CFU/ml	Intra-peritoneum
Group 5	7	Bacterial culture supernatant	Intra-peritoneum
Group 6	4^a^	Bacterial swab	Injury in: head, back, abdomen, leg
Group 7	7	Negative control	None

^a^10^5^CFU/ML, low colony counts 102CFU/ML, High colony counts

#### Behavioral responses of mice

The mice were observed at least twice each day for clinical signs of fatigue, allergies, and aggressiveness.

#### Histological examinations

Infected organs of tested mice were collected and preserved in 10% formalin for histopathological processing for many days. Dehydration was done using 100% alcohols for 20 min and isopropanol for 65 min by rapid microwave histo-processor. The selected tissues were transferred to paraffin wax at a melting point of 2 mm thickness and allowed to cool solidity. Embedded tissues were cut in 5 μm by a rotator microtome. The sections were stained with hematoxylin and eosin (H&E). Sections were fixed on glass slides covered by coverslip [27].

### Statistical analysis

The temperature was analyzed by Microsoft Excel (Microsoft Office). Collected data and data of pathogenicity tests were analyzed by The Statistical Package for Social Sciences (SPSS) program version 23 using chai square. Statistical significance was set at *P* < 0.05, with 95% confidence interval.

## Results

### Affected animals

In this study, 39.10% of donkeys have wounds. Donkeys in Omdurman and Khartoum North were of similar working age (*p* = 0.7), but there was a significant association between area and type of work (*p* = 0.01).

### Primary biochemical test

One hundred and twenty-two samples (122) from wound secretions were investigated bacteriologically. Four samples with no growth. One hundred sixty-nine isolates were purified and recovered with primary biochemical tests. Twenty-three (23) isolates were gram-positive, non-motile, non-sporulated, not hemolytic, catalase-positive, oxidase-positive, oxidation fermentation test negative and aerobically growing.

### Polymerase Chain Reaction (PCR)

The Universal primer of 16S rRNA amplified a product of approximately 550 bp for 10 isolates (15b, 46b, 56a, 69a, 124B, 211, 225, 103B, 151B and 4a). The product fragments were sequenced. A search of homology in the Gene Bank database by BLAST revealed no results.

### Whole-genome sequencing and genomic features of the strain DaniaSudan

The genome sequence of *strain* DaniaSudan consisted of 2.333.512 bp with a 38.1% GC. The final assembly (GCA-003627575.1) contained 75.967 contigs with N50 of 175 bp length. The largest coting assembled was 469.287 bp lengths.

The number of predicted coding sequences (CDS), number of contigs with (PCE_S_), number of subsystems and number of RNAs were 2473, 353, 250 and 58, respectively. One CRISPR was identified.

### Genomic announcement

**Fig. 1:**
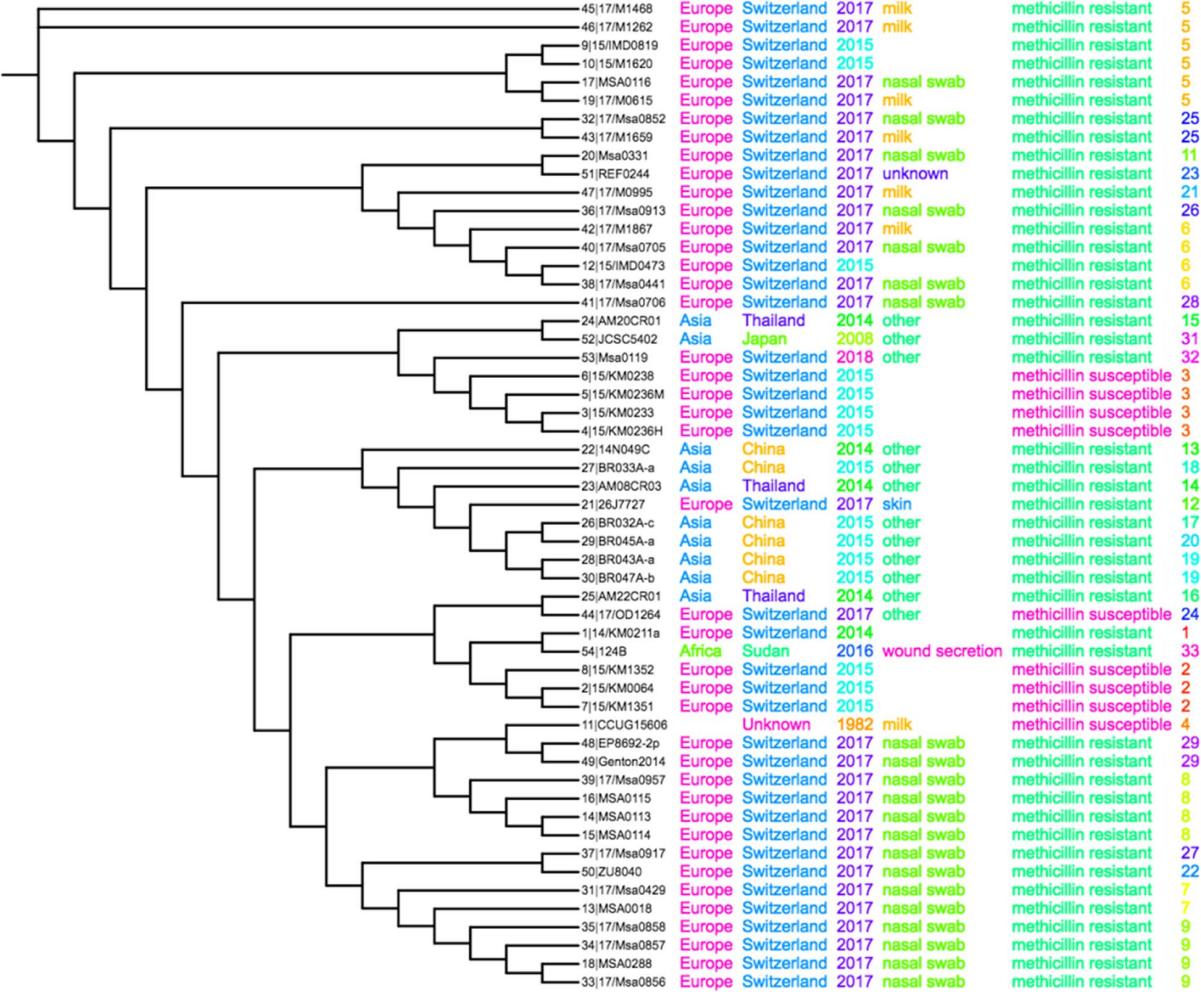
MLST_−_tree_−_V1of strain DaniaSudan. Tree of Methicillin resistant genes fragments examined during multi-locus sequence typing of strain DaniaSudan isolated from external wounds in Sudan

The whole-genome sequence was sent to the center for genomic epidemiology for multi-locus sequence typing (MLST), which identified seven novel alleles: ack-6, cpn60, fdh, pta-1, purA, sar-13 and tuf.8 (Table 2). A phylogenetic tree based on MLST showed relations to strain KM0211a (Fig. 1). Plasmid rep22 was shown in contig 23 by PlasmidFindet-2.0 Server (Table 3). The Number of component sequences (WGS or clone) was 353.

**Table 2:** Allelic profile of strain DaniaSudan as determined by Multi-Locus Sequence Typing-2.0 Server

Locus	Identity	Coverage	Alignment Length	Allele Length	Gaps	Allele
Ack	100	100	400	400	0	ack_−_6
cpn60	98.6207	100	290	290	0	cpn60_−_16
Fdh	98.6667	100	450	450	0	fdh_−_ 10
Pta	98.4091	100	440	440	0	pta_−_1
purA	99.5	100	400	400	0	purA_−_2
Sar	99	100	300	300	0	sar_−_13
Tuf	99.3684	100	475	475	0	tuf_−_8

alleles with less than 100% identity found

cpn60: Novel allele, ST may indicate the nearest ST

Tuf: Novel allele, ST may indicate the nearest ST

Fdh: Novel allele, ST may indicate the nearest ST

Sar: Novel allele, ST may indicate the nearest ST

Pta: Novel allele, ST may indicate the nearest ST

PurA: Novel allele, ST may indicate the nearest ST

**Table 3:** Results of PlasmidFinder-2.0 Server

Plasmid	rep22	rep22
Identity	99.84%	99.84%
Contig	dan_−_contig21	dan_−_contig21
Note	RepB (pAMalpha)	repB (Pvib110)
Accession number	AF503772	X03408

**Fig. 2:**
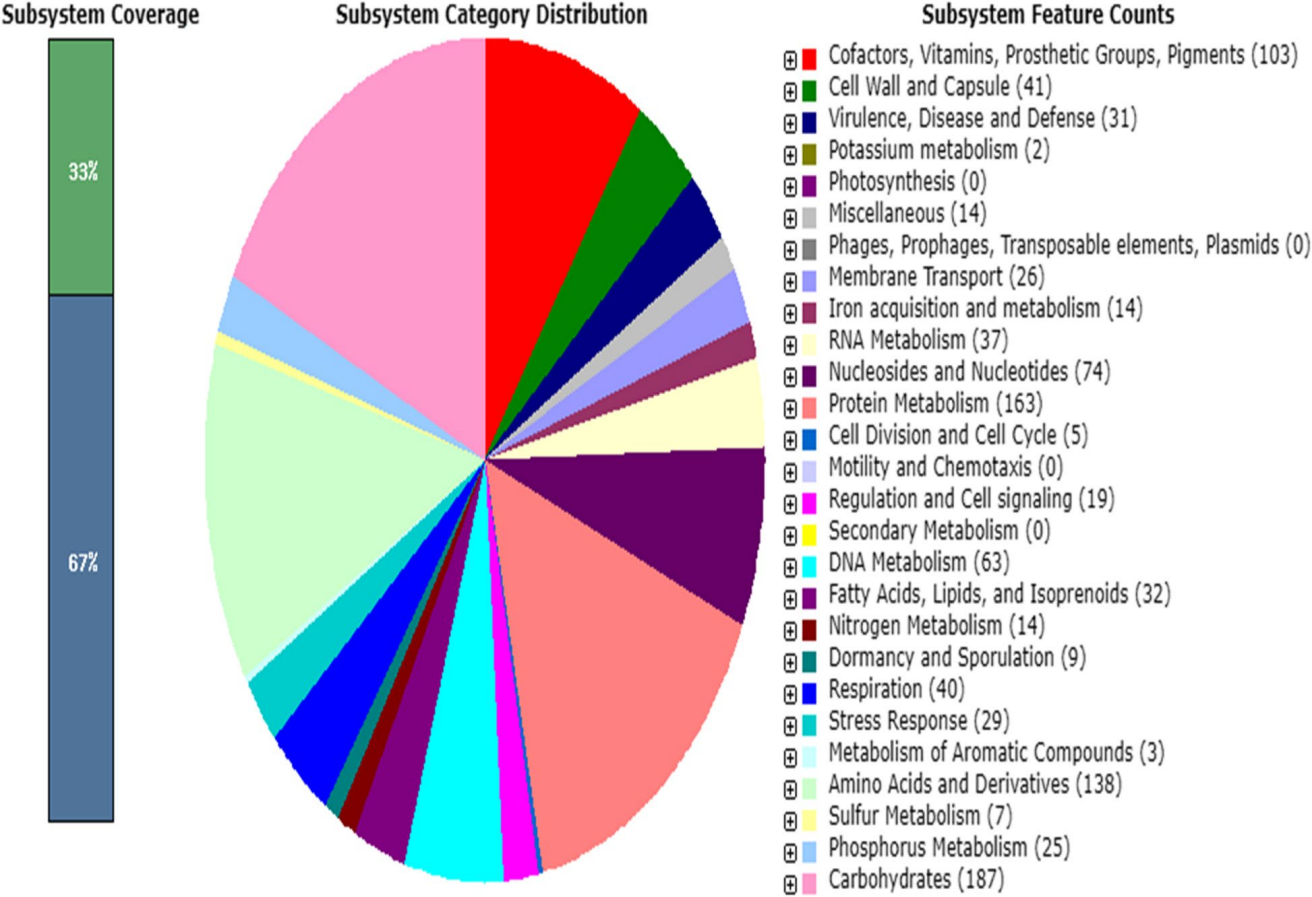
Results of RAST annotation of strain DaniaSudan

Then the sequence was sent to RAST for annotation (Fig. 2). The result of RAST includes many resistance genes methicillin-resistant PBP2 (*mecA, mecI and mec RI*), TatR family (*Tet 38*) and ANT (4′)-Ib (Table 4). The organism has 31 virulence factors of disease and defense.

**Table 4:** Resistance genes of strain DaniaSudan identified by RAST program

Contig	Best Hit ARO	AMR gene family	Identity
1_−_56	Tet(38)	Major facilitator superfamily (MFS) antibiotic efflux pump	99.33%
1_−_ 112	mecI	Methicillin resistant PBP2	99.19%
1_−_ 114	mecA	Methicillin resistant PBP2	99.7%
1_−_ 113	mecR1	Methicillin resistant PBP2	100%
4_−_1	ANT(4′)-Ib	ANT(4′)	99.6%

Annotation was added by the NCBI Prokaryotic Genome Annotation Pipeline (released 2013). https://www.ncbi.nlm.nih.gov/genome/annotation_prok/

### Sequence data access

The whole-genome shotgun project has been deposited at DDBJ/ENA/GenBank under the accession number RBVL00000000. Bio sample SAMN10132107 and Bio project PRJNA493211.

### Phylogenetic analysis of strain DaniaSudan nucleotide

**Fig. 3:**
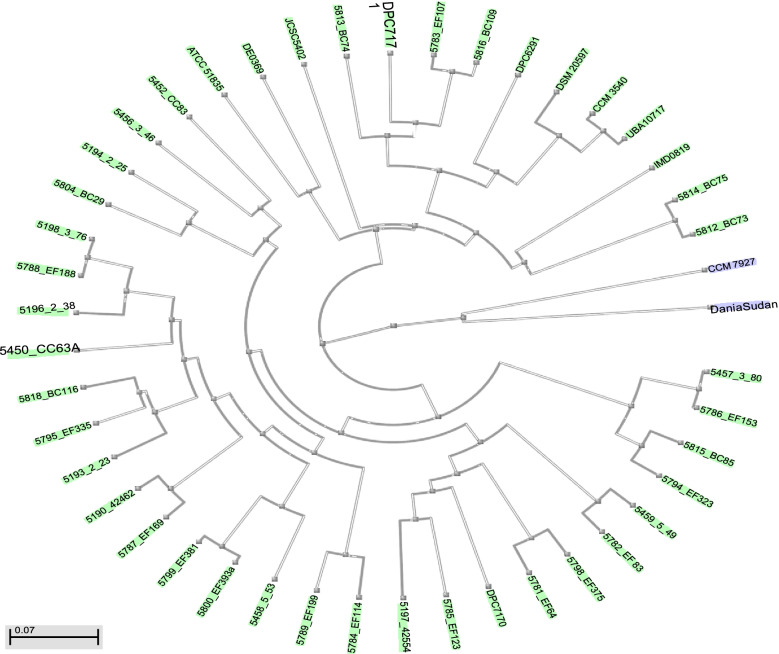
Phylogenetic tree based on 16 rRNA gene sequence showing *M. caseolyticus* strain DaniaSudan and other *Macrococcus* spp. The tree inferred using neighbor-joining method calculated by Kimura’s two-parameter model

Tree Viewer 1.17.5 showed a relationship between the novel strain and *M. caseolyticus* subsp. *hominis* subsp. nov. (type strain CCM 7927 T = DSM 103682 T) (Fig. 3).

### The prevalence of strain DaniaSudan

**Fig. 4:**
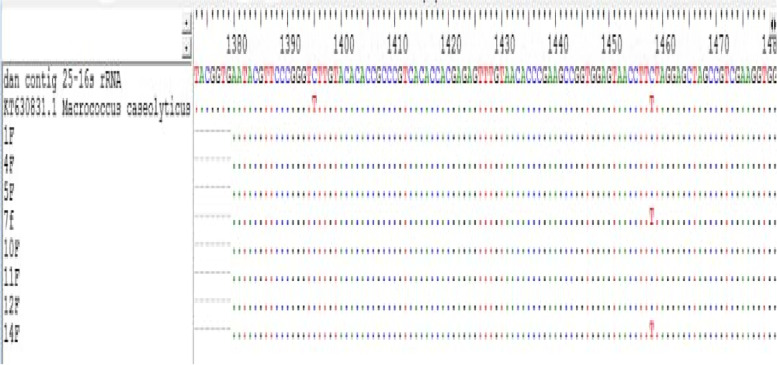
Sequence alignment of *M. caseolyticus* strain DaniaSudan in comparison with sequence of PCR bands by BLAST program

After the isolate 124B was identified as *M. casueolyticus* strain DaniaSudan*,* the 10 bands were alignment with ref. sequence by BLAST program. Isolate 4a, 15b, 56b, 69a, 46b, 211 and 225 were identical to isolate 124B, shown in (Fig. 4). So we have 7 identical isolates to strain DaniaSudan. The prevalence of the strain was 4.73%, with (62.5%) of the isolates collected in winter, (75%) collected from the back of the animal (Table 5).

**Table 5:** *M. caseolyticus* strain DaniaSudan isolates according to animal site, age, study area and season

Isolate	Age	Season of collection	Location of wound	Study area	Duration of work
65b	> 10	Winter	Back	Omdurman	>8hor
124B	< 10	Summer	Back	Omdurman	<8hor
69a	< 10	Winter	Back	Omdurman	>8hor
15b	< 10	Winter	Abdominal	Omdurman	>8hor
211	< 10	Raining	Back	Khartoum north	>8hor
4a	> 10	Winter	Head (eye)	Khartoum north	>8hor
46b	> 10	Winter	Back	Omdurman	>8hor
225	> 10	Raining	Back	Omdurman	>8hor

### Sensitivity test

Eight identified isolates were subjected to antibiotics sensitivity test. The organism was found resistant to ciprofloxacin, ceftazidime, erythromycin, oxacillin, clindamycin and kanamycin. However, the organism was susceptible to imipenem, ampicillin, cefoxitin, trimethoprim/sulphamethoxazole, cephalothin, vancomycin, neomycin, tetracycline and novobiocin and intermediate to penicillin G and chloramphenicol.

### Clinical signs

Clinical signs and temperatures of seven different groups were observed and recorded for 7 days. Mice in the control group did not show any clinical signs during the 7 days.

### Changes in mice temperature

The temperatures of all mice under test were measured before bacterial injected. The temperatures of (G1, G2, G3, G4, G5 and G6) were increased; the mean temperature was 40.9 °C. The highest temperatures were recorded on day five. In contrast, the control group (G7) remained with no change in the temperature during the experimental period.

### Behavioral responses of mice

All injected mice swelling, an allergy, developed wounds were seen with highly significant association *p* = 0.001, *p* = 0.000, *p* = 0.025 respectively. While loss of hair were seen in both (G3 and G4) injected with (10^2^ CFU/ml) s/c and i/p with highly significant association *p* = 0.005. In addition significant results were seen between the site of injection and swelling and loss of hair with significant association *p* = 0.001 and *p* = 0.005.

### Gross lesions

**Fig. 5:**
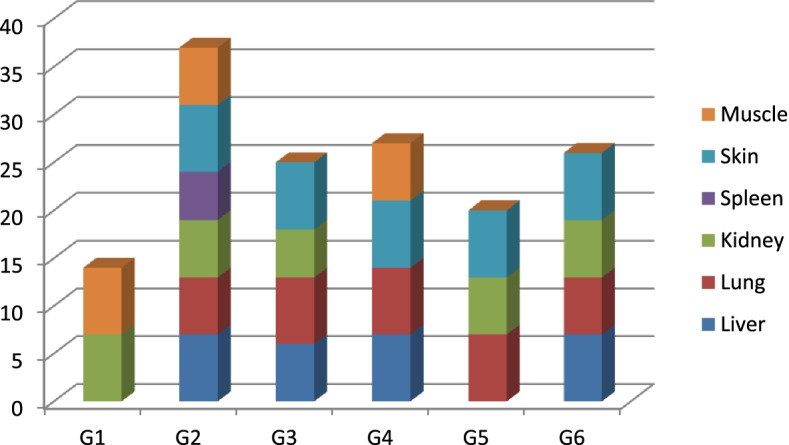
Pathogenic lesions for experimental group

No pathogenic lesions were seen in the control group (G7); however, G1, G2, G3, G4, G5 and G6 have different pathologic lesions, while G2 (10^5^ CFU/ml which injected intra-peritoneum) the lesion seen in liver, lung, kidney, spleen, skin and muscle (Fig. 5).

### Microscopic lesions

Internal organs showed pathological changes, including liver, lung, kidney and spleen Figs. 6, 7, 8, 9, 10, 11 and 12. Moreover, skin and muscle showed pathogenic changes Figs. 13, 14, 15, 16 and 17, respectively.

**Fig. 6:**
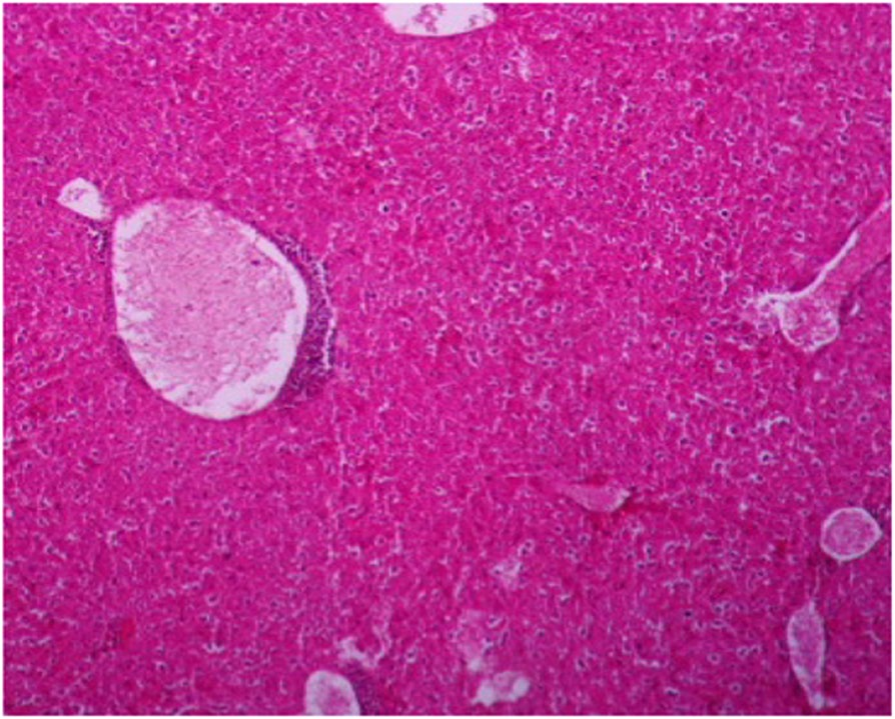
Liver section showing dilated central veins with thrombus formation, infiltration of inflammatory cells around central veins and loss of lobulation (H&E 10X)

**Fig. 7:**
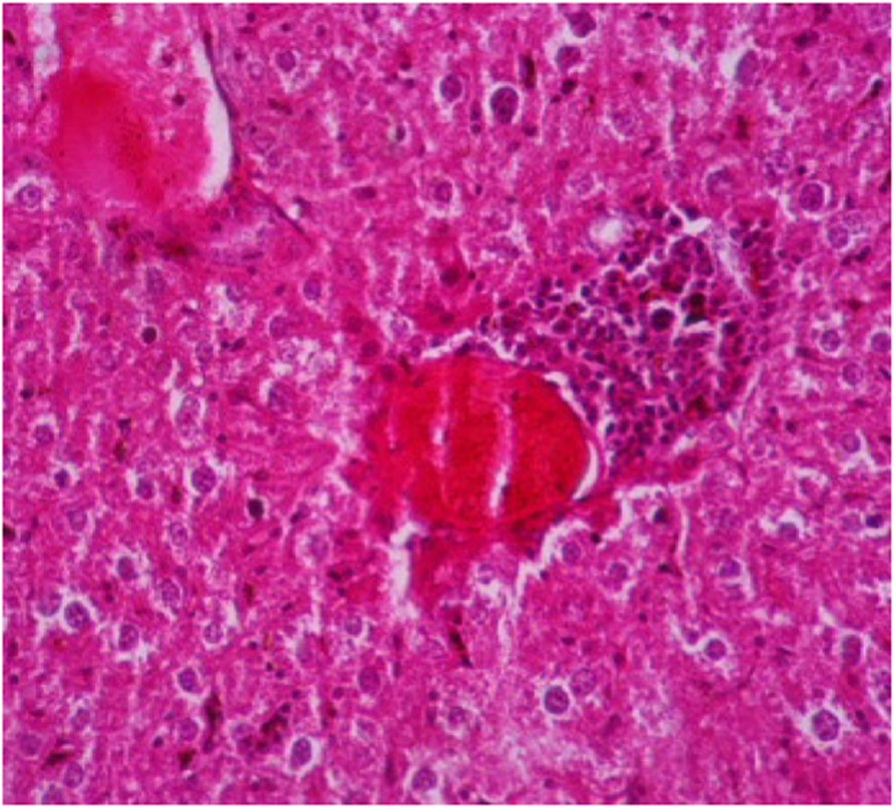
Liver section showing hepatic cells necrosis, central veins are dilated with thrombus formation and infiltration of inflammatory cells around central veins (H&E 40X)

**Fig. 8:**
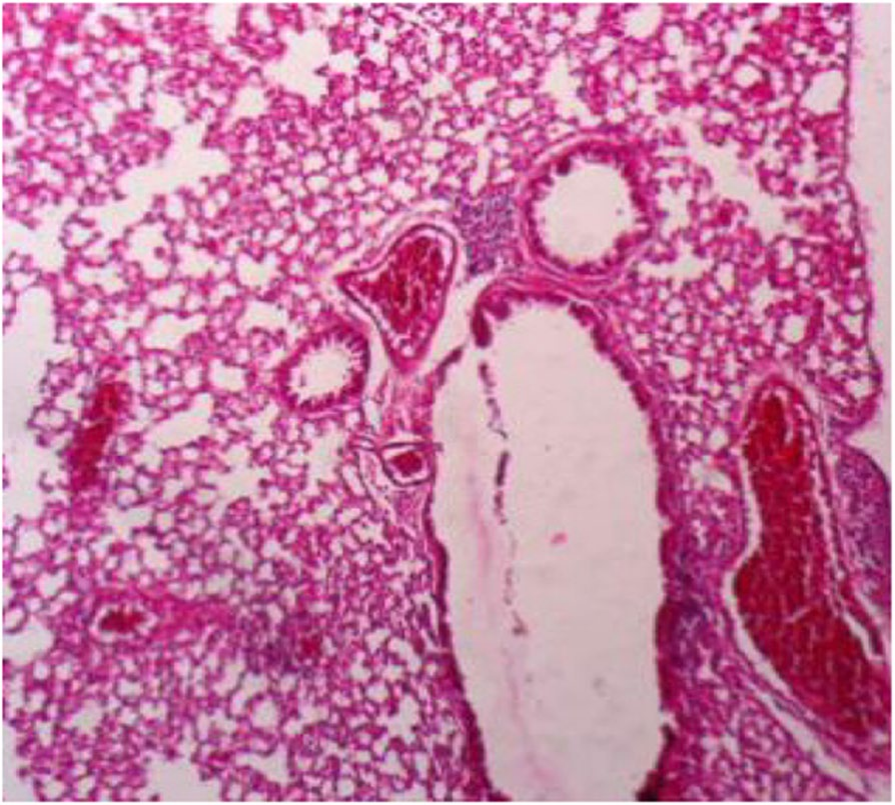
Lung section showing thickening of the alveolar wall (interstitial pneumonia), emphysema, congested blood vessels, dilated bronchioles with necrosis of bronchial epithelium and infiltration of inflammatory cells (H&E 10X)

**Fig. 9:**
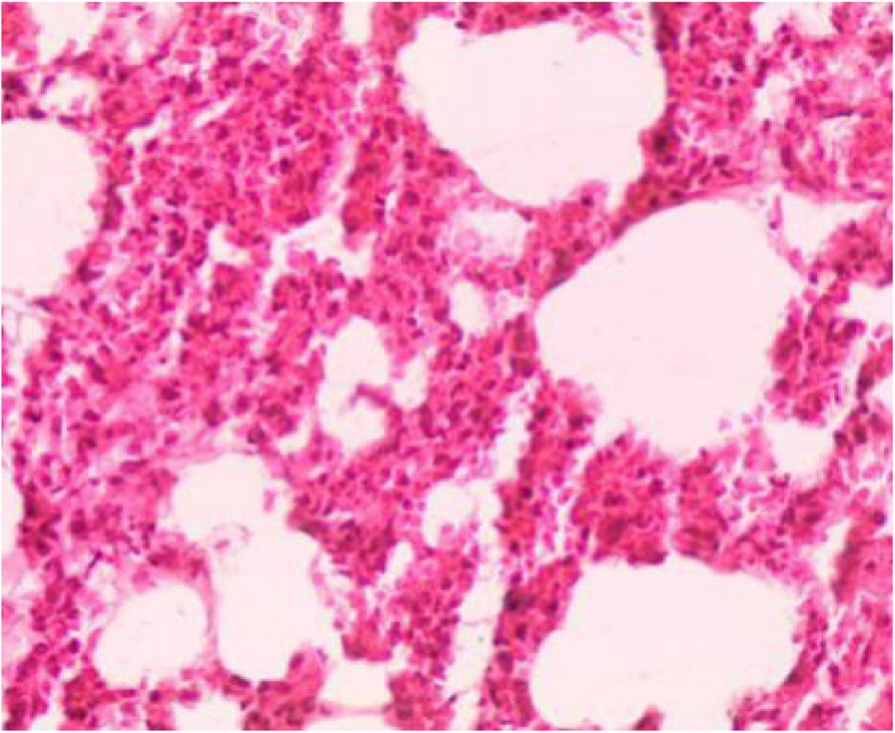
Lung section showing interstitial pneumonia (H&E 40X)

**Fig. 10:**
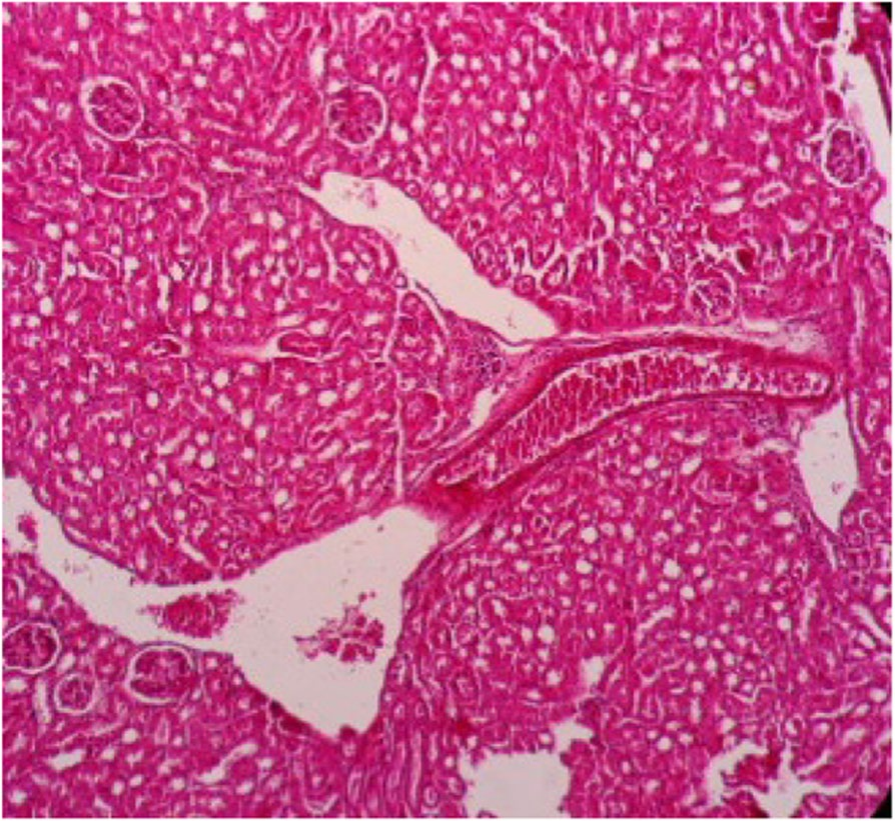
Kidney section showing glomeruli segmentation and polymorphism, necrosis of renal tubules, congestion of blood vessels and heavy infiltration of inflammatory cells (H&E 10X)

**Fig. 11:**
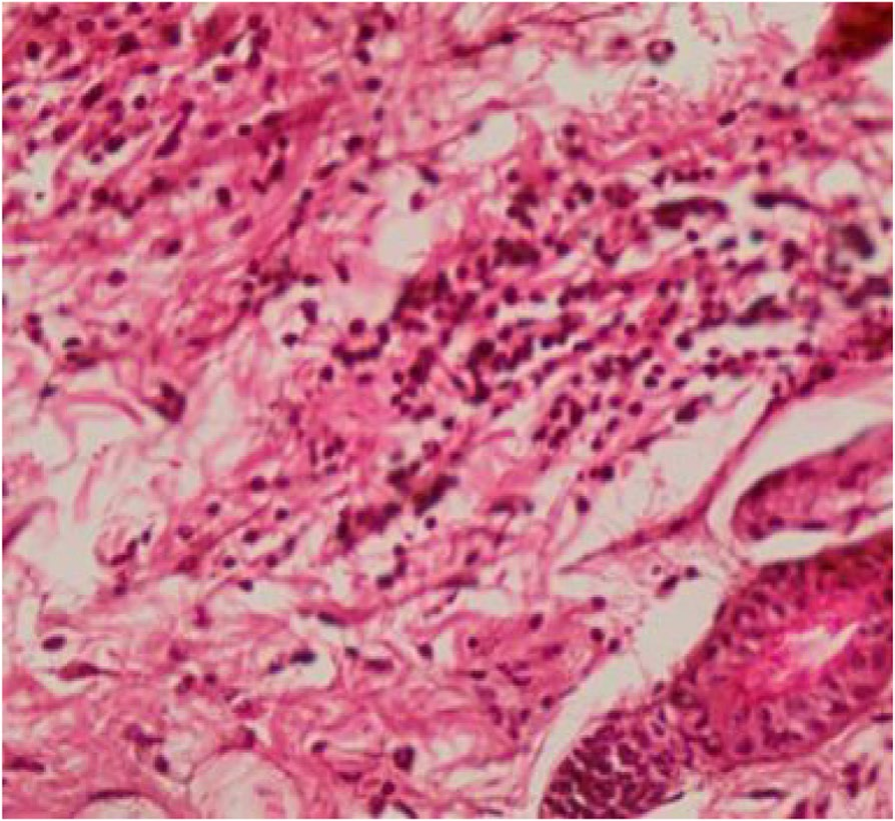
Kidney section showing dilation of glomeruli and segmentation of glomerular tough, hemorrhage and renal tubules were dilated and necrotic (H&E 40X)

**Fig. 12:**
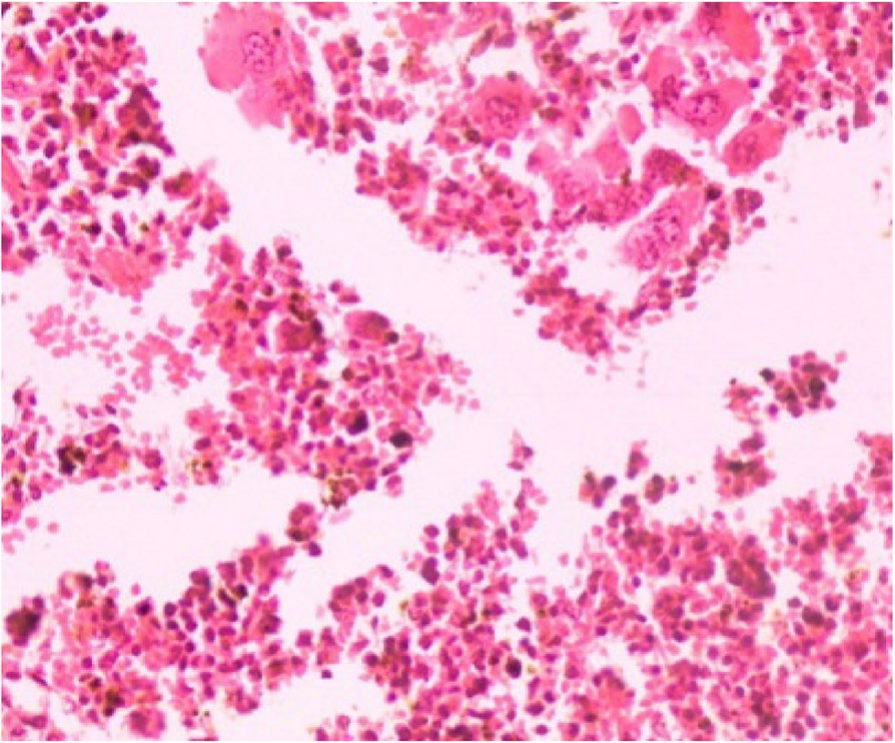
Spleen section showing lymphocytic depletion, increase number of lymphoblast, hemorrhage with deposition of yellowish-brown pigment (indicative of hemosiderin) (H&E 40X)

**Fig. 13:**
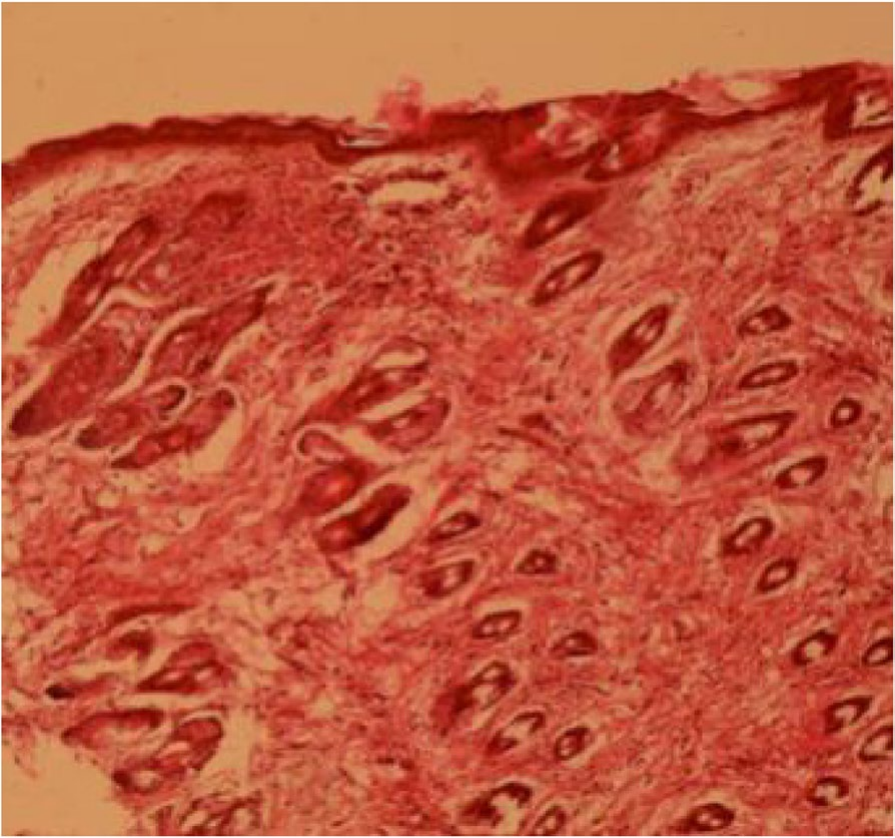
Skin section showing heavy infiltration of inflammatory cells (H&E 10X)

**Fig. 14:**
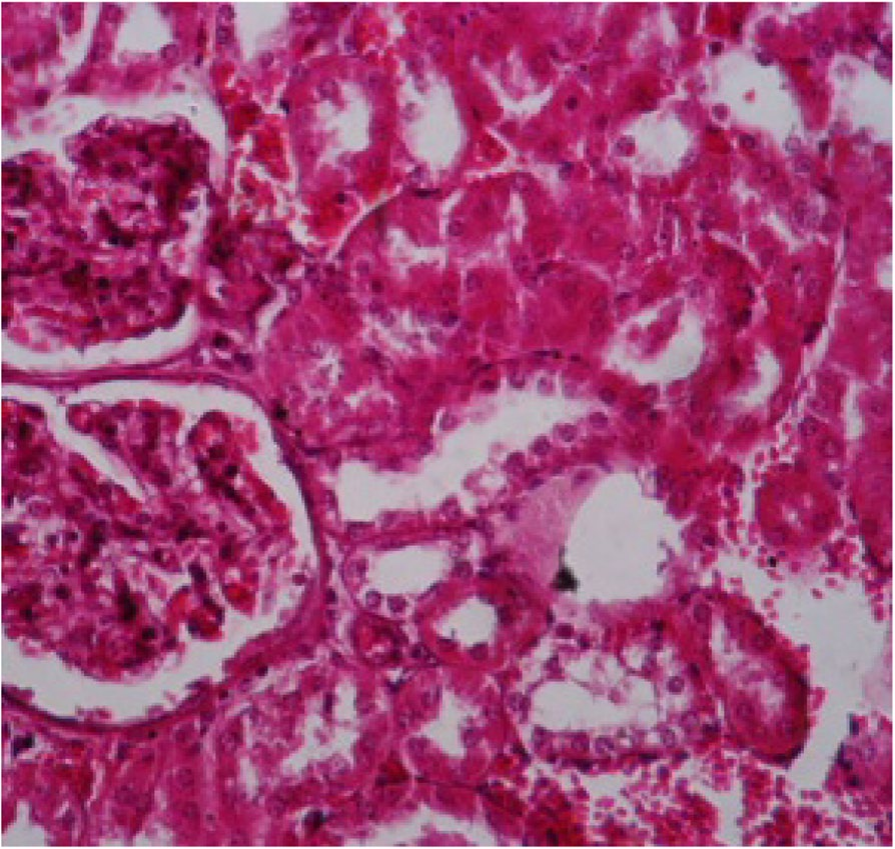
Skin section showing heavy infiltration of inflammatory cells (H&E 40X)

**Fig. 15:**
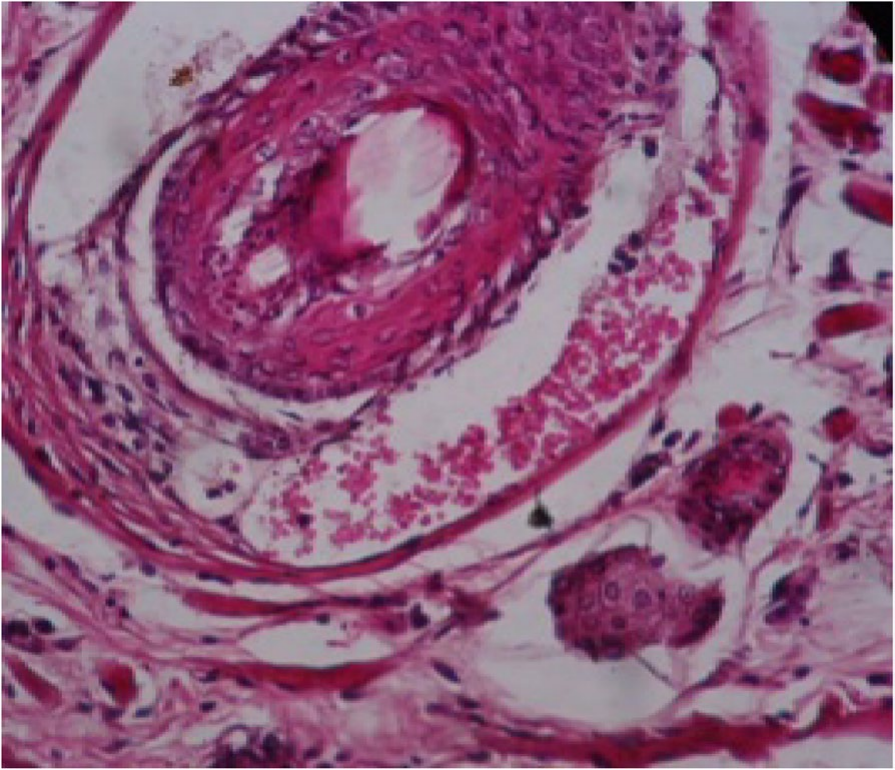
Skin section showing hemorrhage in the hair follicles and infiltration of inflammatory cells (H&E 40X)

**Fig. 16:**
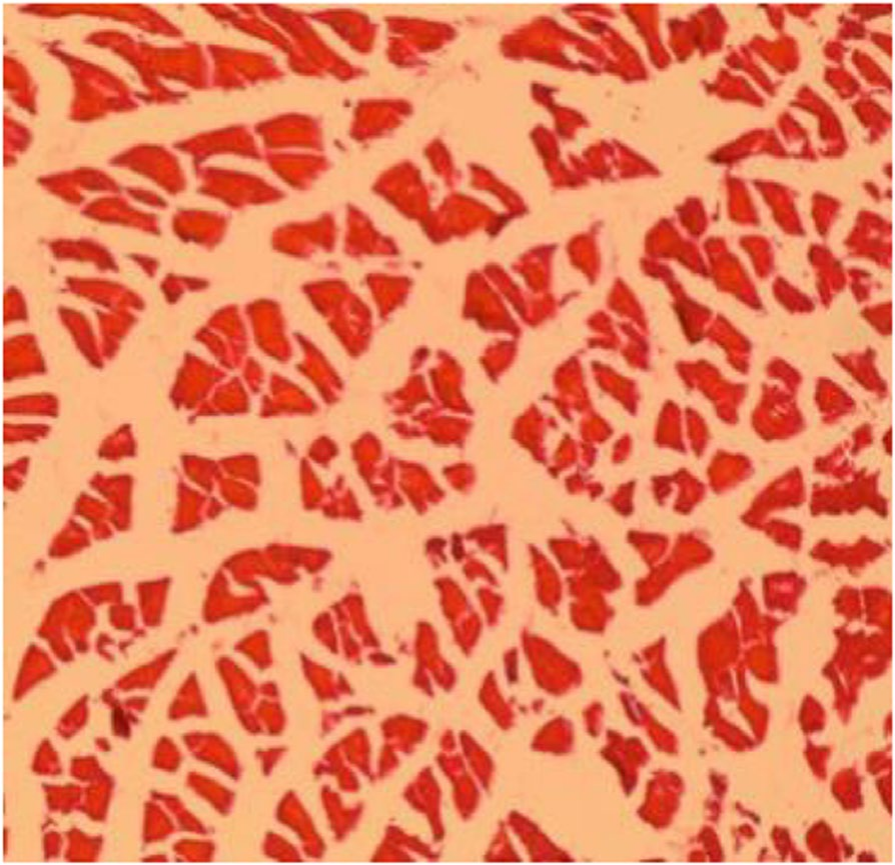
Muscle section showing degeneration of muscle fibers (H&E 10X)

**Fig. 17:**
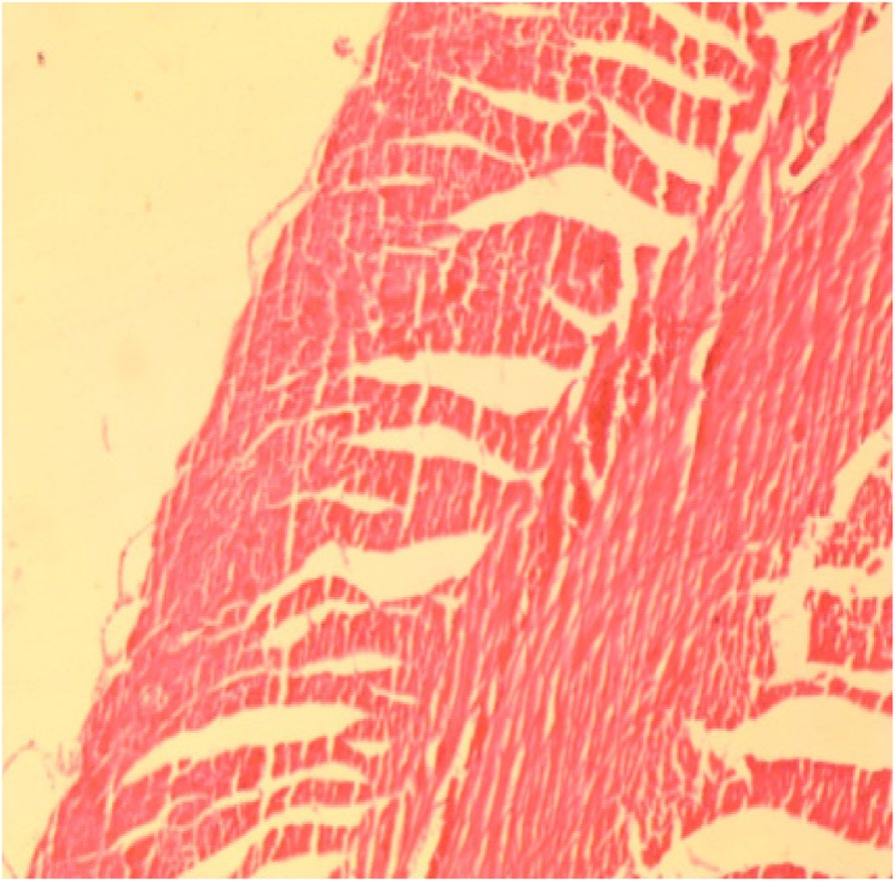
Muscle section showing degeneration of muscle fibers (H&E 40X)

## Discussion

Donkey’s health in Sudan is vital for trade, economy, society and veterinary medicine. Skin diseases adversely affect donkeys’ ability to work. The skin and body surfaces of animals provide a wide area for microbial colonization. Bacteria from those sources seem to be saprophytic or parasitic, but they can also play an important role in various infections [8]. Some skin disorders that affect donkeys in tropical climates are very serious for both donkeys and their owners. Skin diseases in donkeys were rare. Traumatic injuries represent serious complications in many places, and secondary infections of these injuries and other inflammatory disorders were common [28].

*M. caseolyticus* was isolated from wounds of donkeys at selected locations in Khartoum State. The study revealed that 39.10% of studied animals had wounds, whereas a similar study in Ethiopia showed 47.7% [3]. The increase of wounds in summer was due to the heavy work during this season, which can be justified as donkeys mainly transported more water.

The distribution of wounds in different body parts includes back sores, wither sores, mouth-commissure sores, tail-base sores, ribs sores, chest sores and girth sores [6, 29]. The most affected part of the body was the back due to the saddle [3]. There was no significant difference between working age in the State.

The DaniaSudan strain was different from other *M. caseolyticus* strains in the negativity to the oxidation fermentation test. The genomic DNA of *M. caseolyticus* strain and the alignment of DaniaSudan sequence amplified by 16S rRNA revealed negative results by BLAST, so the DNA of sample (124B) was send to whole genome sequencing.

The complete genome sequence of *M. caseolyticus* DaniaSudan has been deposited at DDBJ/EMBL/GenBank under the accession number RBVL00000000 belongs to BioProject PRJNA493211 and BioSample SAMN10132107. The whole-genome revealed that the organism had 354 nucleotides and the protein sequences were 2401. N_50_ 147.392, L_50_ 5, number of contigs with (PEGs) was 353, numbers of coding sequence were 2473 and numbers of RNAs were 58. The genome was notated as a novel strain. The NCBI genome neighbor report showed that *M. caseolyticus* subsp. *hominis* subsp. nov. (type strain CCM 7927 T = DSM 103682 T) and *M. caseolyticus* strain DaniaSudan were displayed 80.3236% symmetric identity and 97.5222% gapped identity with each other.

The prevalence of the strain was 4.73%, and observed that 62.5% of the isolates were recovered in winter when the temperature was the lowest in the state. At the same time, 75% were collected from the back of the animal.

Strain DaniaSudan was resistant to ciprofloxacin, ceftazidime, erythromycin, oxacillin, clindamycin and kanamycin, which was agreed only with the resistance of type strain CCM 7927 T = DSM 103682 T to erythromycin. However, the novel strain was susceptible to imipenem, cefoxitin, cephalothin, tetracycline and novobiocin. While strain type CCM 7927 T = DSM 103682 T was susceptible to ampicillin, cefoxitin, cephalothin, ciprofloxacin, clindamycin, gentamicin, chloramphenicol, imipenem, kanamycin, neomycin, oxacillin, penicillin G, sulfamethoxazole/trimethoprim (cotrimoxazol), tetracycline and vancomycin [30]. The resistances of strain DaniaSudan to oxacillin are due to MRSA (*mecA* gene) in the genome sequence.

All injected mice exhibited some symptoms of toxicity such as slow movement, hair erection and loss of appetite. Increasing of temperatures (fever) of all groups was indicating a bacteremia, which was identified by isolating the organism from the blood. However, injection of supernatant induced high temperature, which could indicate the presence of *M. caseolyticus* toxins in the supernatant.

There was a highly significant association between the dose and swelling (*p* = 0.001), dose and developing of allergy (*p* = 0.000) and a significant association between dose and hair loss (*p* = 0.005). The developing of allergy is similar to the observation of allergy in dogs [31]. There were also a significant association between location of injection and developing of wounds in all groups (*p* = 0.019), this indicated that wounds are syndromes of the infection and considered route of infection.

Skin showed hyperplasia of epidermal laxer, which was more related to histopathological studies of mice by *Staphylococcus spp.* [32] It recognized that epidermal hyperplasia is a reaction to an activated immunity response [33]. A coagulase-positive Staphylococcus species usually cause glandular necrosis and infiltrations of inflammatory cells (folliculitis) in equine [34].

Liver showed congestion in central veins, there was hypertrophy of hepatic cells, necrosis of hepatocytes, nuclei enlarged, vesicular appearance and infiltrations of inflammatory cells. Lung showed hemorrhages, emphysema, edema and thickening of the alveolar wall (interstitial pneumonia), congested blood vessels, dilated bronchioles with necrosis of bronchial epithelium and infiltration of inflammatory cells, which related to infection by Staphylococci [35, 36]. The Kidney showed dilation of the glomerular capsule, polymorphism, necrosis of renal tubules, congestion of blood vessels and heavy infiltration of inflammatory cells observed in infection by Staphylococci and other bacteria. Mainly caused by antibiotic-resistant and bacterial toxins [37, 38]. Spleen showed lymphocytic depletion [39], which is seen in rats infected by *E. coli.* Increase number of lymphoblast, hemorrhage with deposition of yellowish-brown pigment. Sagoh [40] reported that hemorrhage in the spleen is caused by portal hypertension. In the present investigation, the pathogenic role was confirmed with obvious effects on the viability, and induction appearance of various clinical symptoms ending with changes in the livers, kidney and spleen of G2, injected with the higher dose intra-peritoneal.

Increased of body temperature (fever), wounds, deficiency of many protein and mutation of proteins were considered as muscle degeneration [41, 42]. In the study, we suggested the purpose of denegation was the fever and wounds.

However, there was no significant association between injection and pathological changes in the eye; this could be a contamination by tears of infected eyes in donkey and mice.

According to the developed high body temperature (fever), swelling with significant association (*p* = 0.019). Infiltration of inflammatory cells in liver, lung, spleen, kidney, muscle and skin were indicated a systemic infection. However, systemic infection was clearer in G5, which has been injected with supernatant intra-peritoneum. The study revealed that skin the most affected organ, which is near to histology of skin infected by the staphylococci and streptococci [17]. This study is the first one on the pathogenicity of *Macrococcus* spp.

## Conclusion

This study revealed the globally of *M. caseolyticus* strain DaniaSudan in the world. The organism caused bacteremia and other symptoms including, swelling, loss of hair and back, abdomen and head wounds. The organism can be transmitted by injury. Lung, liver, spleen, muscle and skin were infected by strain DaniaSudan, indicating systemic disease. The injury sites of collected isolates were identical to the location of wounds in mice. The virulence factors, CRISPRT and Plasmid in the genome sequence approved the results of mice model.

## Supplementary Information

**Additional file 1.**

**Additional file 2.**

**Additional file 3.**

**Additional file 4.**

**Additional file 5.**

**Additional file 6.**

**Additional file 7.**

## Data Availability

The datasets used and/or analyzed during the current study are available from the corresponding author on reasonable request at (daniaelmahi811@gmail.com).

## Abbreviations

CDS: Coding sequences
MRSA: Multidrug-resistant *Staphylococcus aureus*
NGS: Next-generation sequencing
WGS: Whole-genome sequencing
BLAST: Basic Local Alignment Search Tool
MLST: Multi-locus Sequence Typing
RAST: Rapid Annotation using Subsystem Technology
