# Supplementary material for: A prevalence and molecular characterization of novel pathogenic strains of Macrococcus caseolyticus isolated from external wounds of donkeys in Khartoum State –Sudan
Source: BMC Vet Res. 2022 May 25;18:197. doi: 10.1186/s12917-022-03297-2 (PMC9131596; doi:10.1186/s12917-022-03297-2)
Supplement: Supplementary file 1 — Additional file 1. [file 12917_2022_3297_MOESM1_ESM.docx]

1. The study only involves of collecting of the samples and the raw data without treatment.
2. The study was carried out in compliance with the ARRIVE guidelines.
