# Supplementary figures and images for: A prevalence and molecular characterization of novel pathogenic strains of Macrococcus caseolyticus isolated from external wounds of donkeys in Khartoum State –Sudan
Source: BMC Vet Res. 2022 May 25;18:197. doi: 10.1186/s12917-022-03297-2 (PMC9131596; doi:10.1186/s12917-022-03297-2)

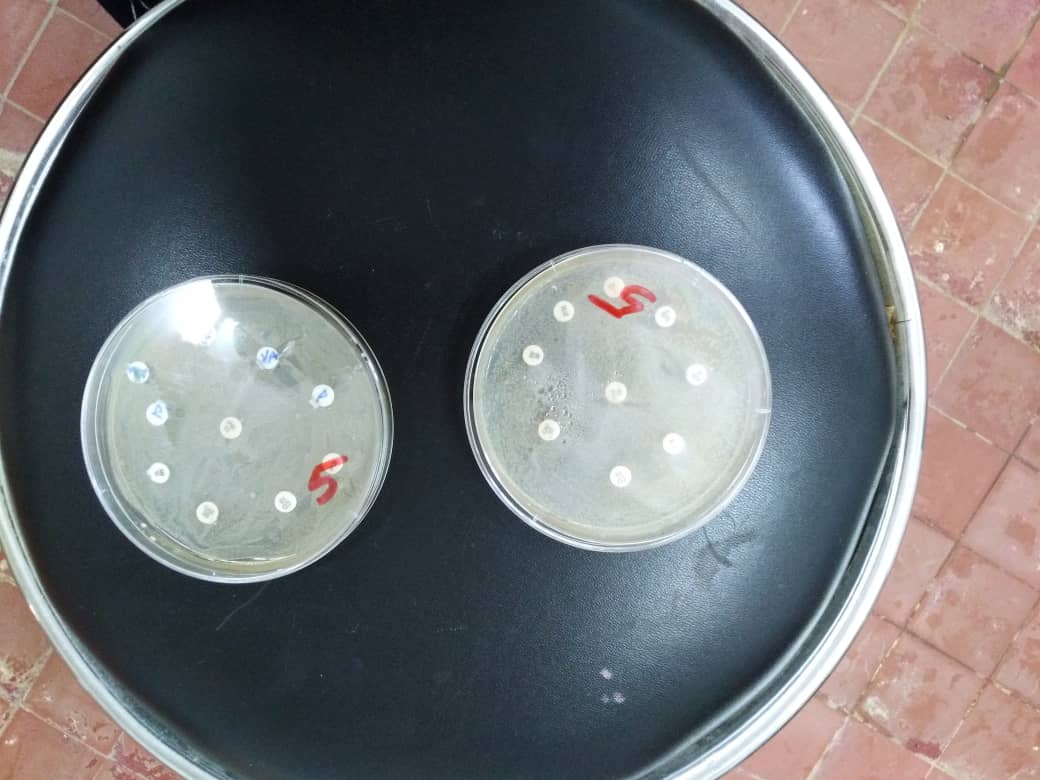

Supplement: Supplementary file 3 — Additional file 3. [file 12917_2022_3297_MOESM3_ESM.jpg]
