# Supplementary material for: A prevalence and molecular characterization of novel pathogenic strains of Macrococcus caseolyticus isolated from external wounds of donkeys in Khartoum State –Sudan
Source: BMC Vet Res. 2022 May 25;18:197. doi: 10.1186/s12917-022-03297-2 (PMC9131596; doi:10.1186/s12917-022-03297-2)

1. Description of data


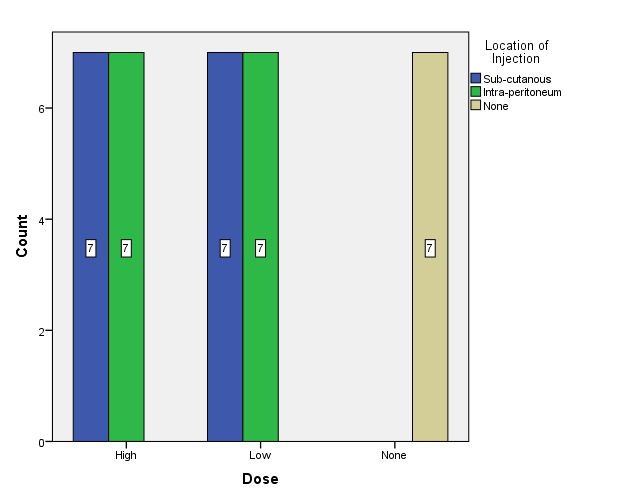


1. **Association between dose and swelling:-**

|  | | | | | |
| --- | --- | --- | --- | --- | --- |
| Dose | | | Swelling | | Total |
|  |  |  | Yes | No |  |
|  | High |  | 9(64%) | 5(36%) | 14 |
|  | Low |  | 12(86%) | 2(14%) | 14 |
|  | None |  | 0(00%) | 7(100%) | 7 |
| Total | |  | 21(60%) | 14(40%) | 35 |

The above table showed that 14 mice have high dose, 9 of 14 ( 64%) were developed swelling and only 5 were not, as well as in the low dose the majority 12(86%) developed swelling with significant association between the dose and swelling p value <0.05(0.001)


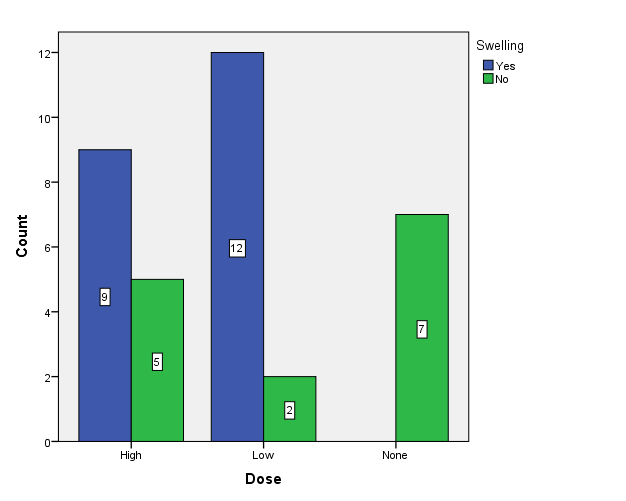


1. **Association between dose and allergy :-**

|  | | | | | |
| --- | --- | --- | --- | --- | --- |
|  | | | Allergy | | Total |
|  |  |  | Yes | No |  |
| Dose | High | Count | 14 | 0 | 14 |
|  |  | % within Dose | 100.0% | 0.0% | 100.0% |
|  | Low | Count | 0 | 14 | 14 |
|  |  | % within Dose | 0.0% | 100.0% | 100.0% |
|  | None | Count | 0 | 7 | 7 |
|  |  | % within Dose | 0.0% | 100.0% | 100.0% |
| Total | | Count | 14 | 21 | 35 |
|  |  | % within Dose | 40.0% | 60.0% | 100.0% |

All of mice’s with high dose have developed an allergy while those with low dose of …… were not. Chi square test revealed that highly significant association between dose and developing of allergy with p value <0.05(0.000).


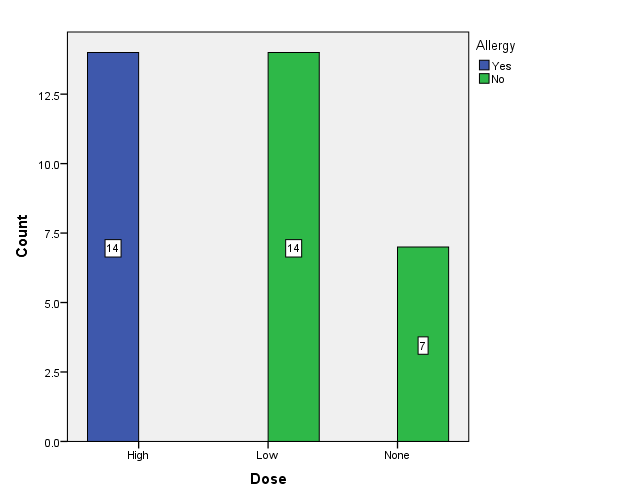


1. **Association between dose and developing of wound**

|  | | | | | |
| --- | --- | --- | --- | --- | --- |
|  | | | Wound | | Total |
|  |  |  | Yes | No |  |
| Dose | High | Count | 8 | 6 | 14 |
|  |  | % within Dose | 57.1% | 42.9% | 100.0% |
|  | Low | Count | 8 | 6 | 14 |
|  |  | % within Dose | 57.1% | 42.9% | 100.0% |
|  | None | Count | 0 | 7 | 7 |
|  |  | % within Dose | 0.0% | 100.0% | 100.0% |
| Total | | Count | 16 | 19 | 35 |
|  |  | % within Dose | 45.7% | 54.3% | 100.0% |

The above table showed that 8 out of 14 mice have develop a wound in both low and high dose and the controls were not

This finding was revealed a significant association between the dose and developing of wound with p value <0.05 (0.025)


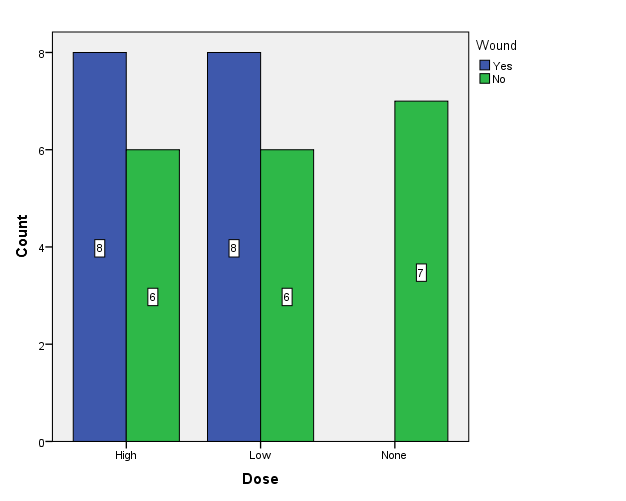


1. **Association between dose and hair lost**

|  | | | | | |
| --- | --- | --- | --- | --- | --- |
|  | | | Hair Lost | | Total |
|  |  |  | Yes | No |  |
| Dose | High | Count | 9 | 5 | 14 |
|  |  | % within Dose | 64.3% | 35.7% | 100.0% |
|  | Low | Count | 10 | 4 | 14 |
|  |  | % within Dose | 71.4% | 28.6% | 100.0% |
|  | None | Count | 0 | 7 | 7 |
|  |  | % within Dose | 0.0% | 100.0% | 100.0% |
| Total | | Count | 19 | 16 | 35 |
|  |  | % within Dose | 54.3% | 45.7% | 100.0% |

In the high dose 9 out 14 mice have loosed their hair while in low dose 10,these result showed signofcant association between dose and hair lost with p value <0.05 (0.005).


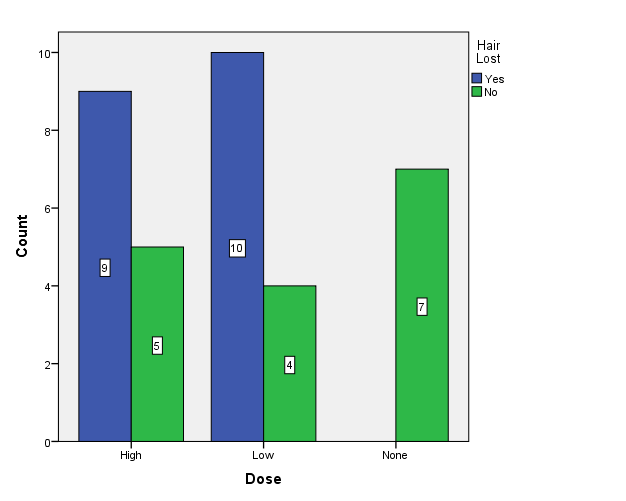


1. Association between dose and change in the eye:-

|  | | | | | |
| --- | --- | --- | --- | --- | --- |
|  | | | Change in the Eye | | Total |
|  |  |  | Yes | No |  |
| Dose | High | Count | 2 | 12 | 14 |
|  |  | % within Dose | 14.3% | 85.7% | 100.0% |
|  | Low | Count | 0 | 14 | 14 |
|  |  | % within Dose | 0.0% | 100.0% | 100.0% |
|  | None | Count | 0 | 7 | 7 |
|  |  | % within Dose | 0.0% | 100.0% | 100.0% |
| Total | | Count | 2 | 33 | 35 |
|  |  | % within Dose | 5.7% | 94.3% | 100.0% |

In the high dose here was 2(….%) of mice’s have changed in their eyes, while in the low dose none of the mice’s were develop with insignificant association between doses and change in the eye with p vale >0.05( 0.204).


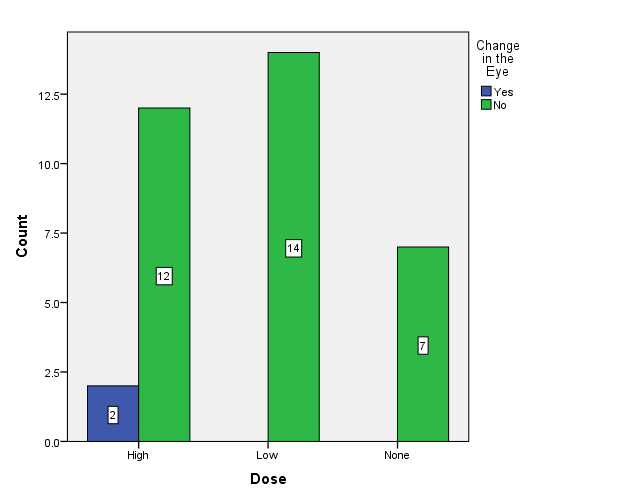


1. Location of injection and swelling

|  | | | | | |
| --- | --- | --- | --- | --- | --- |
|  | | | Swelling | | Total |
|  |  |  | Yes | No |  |
| Location of Injection | Sub-cutanous | Count | 12 | 2 | 14 |
|  |  | % within Location of Injection | 85.7% | 14.3% | 100.0% |
|  | Intra-peritoneum | Count | 9 | 5 | 14 |
|  |  | % within Location of Injection | 64.3% | 35.7% | 100.0% |
|  | None | Count | 0 | 7 | 7 |
|  |  | % within Location of Injection | 0.0% | 100.0% | 100.0% |
| Total | | Count | 21 | 14 | 35 |
|  |  | % within Location of Injection | 60.0% | 40.0% | 100.0% |

Write comment as in the dose

Significant association p value <0.05(0.001)


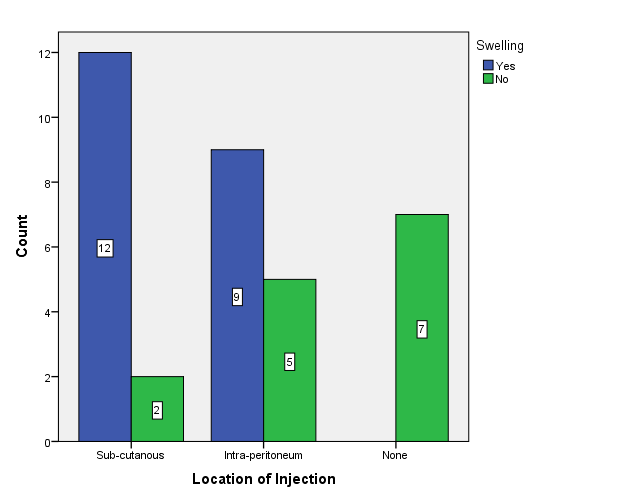


1. Location of injection and allergy:-

| **Crosstab** | | | | | |
| --- | --- | --- | --- | --- | --- |
|  | | | Allergy | | Total |
|  |  |  | Yes | No |  |
| Location of Injection | Sub-cutanous | Count | 7 | 7 | 14 |
|  |  | % within Location of Injection | 50.0% | 50.0% | 100.0% |
|  | Intra-peritoneum | Count | 7 | 7 | 14 |
|  |  | % within Location of Injection | 50.0% | 50.0% | 100.0% |
|  | None | Count | 0 | 7 | 7 |
|  |  | % within Location of Injection | 0.0% | 100.0% | 100.0% |
| Total | | Count | 14 | 21 | 35 |
|  |  | % within Location of Injection | 40.0% | 60.0% | 100.0% |

Insignificant association P value 0.05


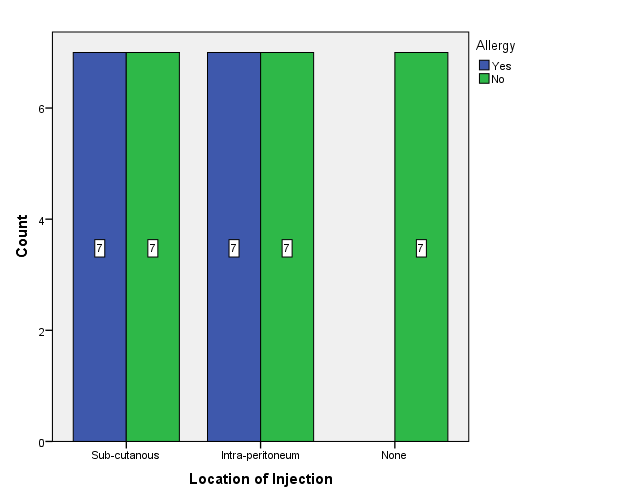


1. Location of injection and wound

|  | | | | | |
| --- | --- | --- | --- | --- | --- |
|  | | | Wound | | Total |
|  |  |  | Yes | No |  |
| Location of Injection | Sub-cutanous | Count | 9 | 5 | 14 |
|  |  | % within Location of Injection | 64.3% | 35.7% | 100.0% |
|  | Intra-peritoneum | Count | 7 | 7 | 14 |
|  |  | % within Location of Injection | 50.0% | 50.0% | 100.0% |
|  | None | Count | 0 | 7 | 7 |
|  |  | % within Location of Injection | 0.0% | 100.0% | 100.0% |
| Total | | Count | 16 | 19 | 35 |
|  |  | % within Location of Injection | 45.7% | 54.3% | 100.0% |

Significant association between location of injection and developing of wound p value <0.05 (0.019).


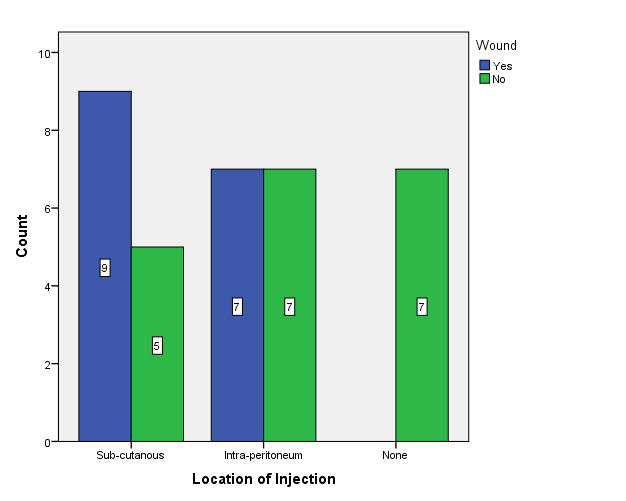


1. Location of injection and hair lost

| **Crosstab** | | | | | |
| --- | --- | --- | --- | --- | --- |
|  | | | Hair Lost | | Total |
|  |  |  | Yes | No |  |
| Location of Injection | Sub-cutanous | Count | 10 | 4 | 14 |
|  |  | % within Location of Injection | 71.4% | 28.6% | 100.0% |
|  | Intra-peritoneum | Count | 9 | 5 | 14 |
|  |  | % within Location of Injection | 64.3% | 35.7% | 100.0% |
|  | None | Count | 0 | 7 | 7 |
|  |  | % within Location of Injection | 0.0% | 100.0% | 100.0% |
| Total | | Count | 19 | 16 | 35 |
|  |  | % within Location of Injection | 54.3% | 45.7% | 100.0% |

Significant association p value <0.05(0.005)


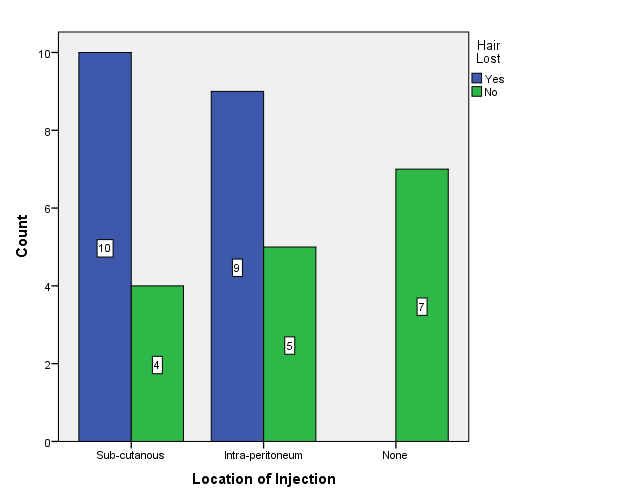


1. Location of injection and change in the eye

| **Location of Injection * Change in the Eye Crosstabulation** | | | | | |
| --- | --- | --- | --- | --- | --- |
|  | | | Change in the Eye | | Total |
|  |  |  | Yes | No |  |
| Location of Injection | Sub-cutanous | Count | 0 | 14 | 14 |
|  |  | % within Location of Injection | 0.0% | 100.0% | 100.0% |
|  | Intra-peritoneum | Count | 2 | 12 | 14 |
|  |  | % within Location of Injection | 14.3% | 85.7% | 100.0% |
|  | None | Count | 0 | 7 | 7 |
|  |  | % within Location of Injection | 0.0% | 100.0% | 100.0% |
| Total | | Count | 2 | 33 | 35 |
|  |  | % within Location of Injection | 5.7% | 94.3% | 100.0% |

Insignificant association between change in the eye and location of injection p value >0.05 (0.204).


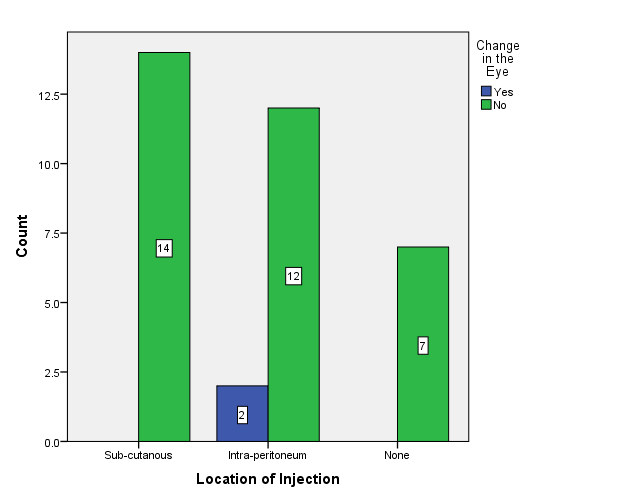

Supplement: Supplementary file 7 — Additional file 7. [file 12917_2022_3297_MOESM7_ESM.docx]
